# Juvenile dermatomyositis in Portuguese children: a case series report

**DOI:** 10.1186/1546-0096-12-S1-P278

**Published:** 2014-09-17

**Authors:** Francisca Aguiar, Joana Abelha-Aleixo, Mariana Rodrigues, Marta D Rosário, Iva Brito

**Affiliations:** 1 Pediatric Rheumatology Unit, São João Hospital, Oporto, Portugal; 2 Rheumatology, São João Hospital, Oporto, Portugal; 3 Paediatrics, São João Hospital, Oporto, Portugal

## Introduction

Juvenile dermatomyositis (JDM) is a rare multisystem disorder that predominantly affects skeletal muscles and skin and less frequently the gastrointestinal tract, myocardium, joints and lungs.

## Objectives

To describe the main epidemiological, clinical and analytical features and outcome of children with JDM followed in our Pediatric Rheumatology Unit**.**

## Methods

Retrospective review of the clinical records of patients with JDM attending our centre since January 2004.

## Results

Seven patients were included, five of them were females. The median age at diagnosis was 10 years (range 4 – 17 years) and the median duration of signs and symptoms was 7 months (range 2 weeks – 2 years). All patients had skin alterations at presentation, which included gottron papules (6/7), heliotrope rash (4/7), raynaud phenomenon (2/7), *livedo reticularis* (1/7) and vasculitis (1/7). Common initial manifestations were also muscle weakness (5/7), myalgia (5/7), constitutional symptoms (5/7), arthralgia (5/7) and arthritis (4/7). One patient presented with cutaneous calcinosis, another one had esophageal involvement and presented with dysphagia and dysphonia. All patients had elevated serum muscle enzymes, four had positivity for antinuclear antibodies and one for rheumatoid factor. Electromyography was done in all patients and was abnormal in five of them; muscle biopsy was done in two patients, cutaneous biopsy in one and magnetic resonance imaging in two, and the results showed changes compatible with dermatomyositis. All patients were initially treated with corticosteroids (n=7), to which it was added, alone or in combination: methotrexate (n= 5), hydroxychloroquine (n=2), azathioprine (n=1) and intravenous immunoglobulin (n=1)**.** The median follow-up time was 4 years (range 4 months – 10 years). One patient developed interstitial lung disease and another one had salivary calcinosis with parotiditis. One patient attained prolonged remission off medication after two years, five patients achieved remission on treatment in a median time of 2 years (range 4 months – 2 years), and one patient had partial response.

## Conclusion

Although this is a small series it indicates that, if diagnosed early and treated adequately, JDM has a good outcome. The most frequent initial manifestations were cutaneous, musculoskeletal and constitutional. During follow-up one patient had gastrointestinal involvement, one developed pulmonary manifestations and other one had salivary calcinosis. Overall there were few complications and a satisfactory response to therapy.

## Disclosure of interest

None declared.

